# Validation of the shortened Hunter Syndrome-Functional Outcomes for Clinical Understanding Scale (HS-FOCUS)

**DOI:** 10.1186/s12955-018-1006-8

**Published:** 2018-11-08

**Authors:** Maria Mattera, Margaret K. Vernon, Mireia Raluy-Callado, Jaromir Mikl

**Affiliations:** 1 Evidera, 7101 Wisconsin Avenue, Suite 1400, Bethesda, MD 20814 USA; 2 Evidera, Metro Building, 6th Floor, 1 Butterwick London, W6 8DL, London, UK; 3 Shire, 300 Shire Way, Lexington, MA 02421 USA

**Keywords:** Hunter Syndrome Functional Outcomes shortened instrument

## Abstract

**Background:**

The rare disease, Hunter Syndrome (mucopolysaccharidosis type II; MPS II), characterized by iduronate-2-sulfatase deficiency, has heterogeneous symptoms often including cognitive impairment (CI). To evaluate physical functioning and daily activity limitations of patients with MPS II, the multidomain shortened Hunter Syndrome-Functional Outcomes for Clinical Understanding Scale (HS-FOCUS) questionnaire was previously developed and preliminarily validated. Here we gather data in a dedicated prospective longitudinal observational study using direct responses to the shortened instrument and assess its psychometric properties further.

**Methods:**

Interview data were collected from eligible self-reporting patients (≥ 12 years of age) or caregivers of patients using respective versions of the instrument at baseline and 2–4 weeks later. Internal consistency, test–retest reliability, convergent and discriminant validity, and validity of known groups were assessed. Participants also completed Child Health Questionnaire (CHQ), Health Utilities Index Mark 3, and Global Impression of Severity (GIS) questionnaires.

**Results:**

All patients were male, consisting of 31 caregiver-reported patients (aged 3–26 years) and 20 self-reported patients (aged 12–58 years). Most (77.4%) caregiver-reported patients had CI. Both questionnaire versions demonstrated good internal consistency and test–retest reliability; Cronbach’s alpha and intra-class correlation coefficients were > 0.70. Spearman’s correlations demonstrated good convergent validity with moderate (> 0.3) to high (> 0.6) correlations of the HS-FOCUS total score with physical functioning, role/social–physical, and bodily pain domains of CHQ. The tool also differentiated between MPS II severity levels based on GIS scores.

**Conclusions:**

The shortened HS-FOCUS questionnaire was found to be a valid and reliable tool to assess the physical functioning impact of MPS II.

## Background

Hunter Syndrome (mucopolysaccharidosis type II; MPS II), one of seven known MPS types [1], is a rare X-linked inherited disease affecting 1 in 170,000 live births. It is characterized by deficiency of the iduronate-2-sulfatase enzyme [2]. Multiple organs can be progressively affected by the disease’s somatic manifestations. The broad spectrum of phenotypes includes skeletal deformities, joint stiffness, hepatosplenomegaly, and heart disease [3], and symptoms can be attenuated (without central nervous system [CNS] involvement) or severe (with CNS involvement), and with or without cognitive impairment (CI). Although genotype/phenotype correlations cannot determine the proportion of patients with CNS involvement, published case series data indicate that 67–78% of patients have the severe or intermediate phenotype [4–7].

Several patient-reported outcome (PRO) tools can be applied to patients with MPS II or their caregivers to assess the impact of the disease on their physical functioning. The Child Health Questionnaire (CHQ) is a generic questionnaire measuring 12 and 14 unique physical and psychosocial concepts in the children’s version (CHQ-CF87) and the caregiver-completed version (CHQ-PF50), respectively [8]. The Health Utility Index Mark 3 (HUI3), existing as self- or parent−/caregiver-reported questionnaires, is part of a group of measures of health-related quality of life and utility (preference) scores. The HUI3 consists of eight attributes (vision, hearing, speech, ambulation, dexterity, emotion, cognition, and pain) [9]. The Global Impression of Severity (GIS) scale consists of a single question that asks the participant to rate the severity of the disease on behalf of themselves or of the patient [10].

The Hunter Syndrome-Functional Outcomes for Clinical Understanding Scale (HS-FOCUS) multidomain questionnaire was designed to evaluate the limitations in physical functioning and daily activities of patients with MPS II. Williams and colleagues highlighted that quality of life and functional assessments were important to identify whether orthopedic surgery is beneficial for some physical aspects of MPS such as carpal tunnel syndrome [11, 12]. Unlike the aforementioned PRO tools, the HS-FOCUS was specifically designed for the assessment of MPS II outcomes and consisted of a patient- and caregiver-reported questionnaire [13]. The original versions of the HS-FOCUS instrument was a longer version consisting of 68 items in the caregiver-reported version and 54 items in the patient-reported version [13].

However, as the fatigue of respondents during a survey may influence their quality-of-life responses, a subsequent study was conducted to test whether a shortened HS-FOCUS questionnaire would increase the efficiency of the instrument and decrease the burden on the respondent [2]. A shortened HS-FOCUS instrument was developed, comprising a patient-reported version (consisting of 32 items) and a caregiver-reported version (consisting of 35 items) [2]. In this study, Wiklund et al. (2014) validated the shortened questionnaire using patient response data from a separate Phase II/III clinical trial (NCT00069641) that incorporated the original long version of the questionnaire [2]. Validation data were not derived directly from responses to the shortened instrument, but rather from responses to the original full-length HS-FOCUS.

The objective of this study was to collect new data in a dedicated prospective longitudinal observational study using direct patient/caregiver responses to the shortened HS-FOCUS questionnaire and to further assess its psychometric properties.

## Methods

### Study design and participant criteria

This was a prospective, longitudinal, noninterventional, observational survey study. Data were collected from self-reporting patients with MPS II (≥ 12 years of age) and caregivers of patients with MPS II (any age) using the respective versions of the shortened HS-FOCUS questionnaire. Eligible participants were recruited from December 2014 to September 2015 through the MPS society in the United States (US) and the United Kingdom (UK) through email outreach, advertisements posted on websites, and in newsletters, and at the MPS Society 28th Annual Family Conference held on December 18–20, 2014 in Orlando, FL.

Informed consent or assent was obtained from all individual participants included in the study. This study was conducted according to US and UK guidelines for the protection of human research subjects and a protocol that was approved by an institutional review board.

Depending on the patient’s ability to complete the questionnaire satisfactorily on their own based on the severity of their disease, patients ≥ 12 years of age completed the self-reported version of the questionnaire. The caregiver-reported version was used when patients were aged < 12 years and/or were unable to complete the questionnaire themselves. All participants were asked to complete written questionnaires at initial assessment and at a follow-up assessment occurring 2–4 weeks later. Both initial and follow-up assessments were delivered to and returned by self-reported and caregiver-reported participants via mail (FedEx). Participants were not instructed to avoid keeping a record of their answers to their initial assessment, but as the answers were hand-written and then delivered via mail rather than email, it is unlikely that the participants kept a record of their assessments.

To evaluate CNS involvement and CI, there were two separate questions in the questionnaires; the first asked the patient/caregiver what type of MPS II the patient had (severe [with CNS involvement] or attenuated [without CNS involvement]), and a second question asked whether the patient had CI (classified as mild, moderate, severe, or very severe).

The questionnaire’s items were grouped into five domains: walking/standing (lower body), grip/reach (upper body), school/work, activities, and breathing. HS-FOCUS scores for each item range from 0 (being able to perform the activity without any difficulty) to 3 (being unable to do so). If less than half of the item responses were missing or not applicable, average domain scores were computed from them. The final HS-FOCUS total score was derived as the mean of individual completed domain scores. A high HS-FOCUS total score was indicative of less physical functioning or lower functional status in patients with MPS II.

Summary statistics of the HS-FOCUS total scores by self- or caregiver-reported version were assessed. In addition, the mean total HS-FOCUS by MPS II disease severity (based on the validated GIS [10] instrument) and self-reported patients were stratified by age group (i.e., 12–17 vs 18–58 years of age).

The shortened HS-FOCUS tool was validated for internal consistency, test–retest reliability, convergent and discriminant validity, and validity of known groups described below.

### Internal and test–retest reliability

Item- and scale-level descriptive statistics were performed to assess the reliability of the shortened instrument. Cronbach’s alpha was used to estimate the internal consistency reliability of the measure, where alpha > 0.70 was considered acceptable [14, 15].

The intraclass correlation coefficient (ICC) was used to determine the test–retest reliability of the tests. For patients considered to be stable (in terms of their disease severity classified as “not at all severe,” “mild,” “moderate,” “severe,” or “extremely severe”) based on GIS, an ICC > 0.70 was considered to be reliable.

### Content validity

Spearman’s rank correlation tests of the shortened HS-FOCUS total scores with domain scores of other questionnaires (patient and caregiver versions) were performed to analyze the convergent and discriminant validity between similar and dissimilar constructs, respectively. Spearman rank correlations between HS-FOCUS total scores at the initial assessment and those determined by CHQ and HUI3 domains were assessed. The CHQ tool was chosen for this analysis, as CHQ measures unique physical and psychosocial concepts [8], and the shortened HS-FOCUS evaluates limitations of physical functioning and daily activities of patients with MPS II. HUI3 was also chosen for this analysis, as this health-related quality of life measurement includes physical attributes of ambulation and dexterity [9]. The statistical significance of calculated Spearman rho correlation coefficients were determined. Analysis of variance (ANOVA) was used to determine the validity of known groups by comparing the mean of the shortened HS-FOCUS total scores with five levels of disease severity based on GIS. For GIS, these severity levels were rated as “not at all severe,” “mild,” “moderate,” “severe,” or “extremely severe” [10]. GIS, CHQ, and HUI3 assessments were performed at initial assessment and at the follow-up assessment, 2–4 weeks later.

All analyses were conducted using the software SAS version 9.4 (SAS Institute, Inc., Cary, NC).

## Results

### Demographics and patient characteristics

Patient demographics and baseline characteristics are summarized in Table 1. The study included 31 caregiver-reported male patients and 20 self-reported male patients (Table 1). The caregiver- and self-reported patients had mean (range) ages of 10.8 (3–26) years and 22.5 (12–58) years, respectively. Seven of the self-reported patients were adolescents and had a mean age of 14.0 (12–17) years. CI involvement was reported by the patient or caregiver rather than by clinical input. Most (77.4%) caregiver-reported patients were reported to have CI involvement, and one member of the self-reported group noted having CI (Table 1). The most commonly reported comorbidities in caregiver- and self-reported patients were carpal tunnel syndrome (61.3% and 75.0%) and cardiac disease (54.8% and 55.0%). Caregiver-reported patients had a higher proportion of psychosocial problems (45%) compared with self-reported patients (10%; Table 1).

**Table 1:** Demographics and patient characteristics of caregiver- and self-reported patients at baseline

Characteristic	Caregiver-reported patients (3–26 years) *n* = 31	Self-reported patients (12–58 years) *n* = 20
Age category, *n* (%)		
3 to < 12 years	17	0
≥ 12 years	14	16^b^
Age in years, mean (SD)	10.8 (6.0)	22.5 (11.4)
Male, *n* (%)	31 (100)	20 (100)
Race, *n* (%)^a^		
White	27 (87.1)	16 (80.0)
Black/African American	4 (12.9)	–
Unknown	–	4 (20.0)
With MPS-affected siblings, *n* (%)	3 (9.7)	4 (20.0)
Age at diagnosis in years, mean (SD)	2.7 (1.5)	4.6 (3.0)^c^
Type of MPS II		
Severe (with CNS involvement)	20 (64.5)	1 (5)
Attenuated (without CNS involvement)	10 (32.3)	15 (75)
Unknown	1 (3.2)	4 (20.0)
Comorbidities, *n* (%)		
Cachexia	1 (3.2)	0
Cardiac disease	17 (54.8)	11 (55.0)
Carpal tunnel syndrome	19 (61.3)	15 (75.0)
Convulsions	2 (6.5)	0
Diarrhea	7 (22.6)	1 (5.0)
Neurodegeneration	6 (19.4)	1 (5.0)
Psychosocial problems	14 (45.2)	2 (10.0)
Severity of CI, *n* (%)	24 (77.4)	1 (5.0)^d^
Mild	4 (16.7)	0
Moderate	9 (37.5)	1 (5.0)
Severe	11 (45.8)	0

^a^Not mutually exclusive

^b^Four patients have missing information

^c^16 patients provided information about their age at diagnosis

^d^CI and its severity are not clinically derived and are reliant on the responses of patients or caregivers

*Abbreviations*: *CI* cognitive impairment, *CNS* central nervous system, *MPS II* mucopolysaccharidosis II, *SD* standard deviation

### HS-FOCUS total scores by patient age category and MPS II severity

Table 2 shows calculated mean total scores of the shortened HS-FOCUS questionnaire by age category for caregiver- and self-reported patients measured at initial assessment and at follow-up. As expected, the mean total HS-FOCUS scores were higher for caregiver-reported patients than for self-reported patients, particularly for adult patients. The mean total scores for adults and pediatric or adolescent patients were similar for self-reported patients; however, the mean total HS-FOCUS score for adult caregiver-reported patients was approximately double that of pediatric or adolescent caregiver-reported patients. Table 2 also presents calculated mean total scores of the shortened HS-FOCUS by MPS II severity. The HS-FOCUS mean scores among caregiver-reported patients with MPS II and CNS involvement were over two-fold higher at both initial assessment and at follow-up, indicating lower functional status, when compared with patients without CNS involvement. Only one self-reporting patient indicated CNS involvement, making comparison of mean HS-FOCUS score by CNS involvement status infeasible for this patient group.

**Table 2:** HS-FOCUS mean total scores by age and MPS II severity for caregiver- and self-reported patients

		Caregiver-reported patients (3–26 years)		Self-reported patients (12–58 years)	
		Initial assessment at baseline	Follow-up at 2–4 weeks	Initial assessment at baseline	Follow-up at 2–4 weeks
Age category					
Pediatric/adolescent	Patients, *n*	27	24	7	5
	Mean (SD) total score	0.92 (0.66)	0.80 (0.51)	0.50 (0.52)	0.45 (0.62)
Adult	Patients, *n*	4	4	13	13
	Mean (SD) total score	2.21 (0.67)	1.95 (0.76)	0.53 (0.67)	0.47 (0.51)
MPS II severity					
With CNS involvement	Patients, *n*	20	18	1	1
	Mean (SD) total score	1.35 (0.81)	1.20 (0.72)	2.1 (−)	1.55 (−)
Without CNS involvement	Patients, *n*	10	9	15	14
	Mean (SD) total score	0.55 (0.43)	0.51 (0.26)	0.41 (0.53)	0.44 (0.50)

*Abbreviations*: *CNS* central nervous system, *HS-FOCUS* Hunter Syndrome-Functional Outcomes for Clinical Understanding Scale, *MPS II* mucopolysaccharidosis II, *SD* standard deviation

### Reliability of the shortened HS-FOCUS questionnaire

Table 3 shows internal consistency results at initial and follow-up assessments as measured by Cronbach’s alpha estimates for HS-FOCUS total scores. For both the caregiver- and self-reported shortened HS-FOCUS questionnaires, Cronbach’s alpha estimates for the HS-FOCUS total scores demonstrated high internal consistency reliability at the initial and follow-up assessments (greater than 0.70, the set criterion for good reliability). For caregiver-reported patients, alpha was 0.89 at the initial assessment (*n* = 25) and 0.86 at follow-up (*n* = 25). For self-reported patients, alpha was 0.94 at the initial assessment (*n* = 12) and 0.92 at follow-up (*n* = 13). Similarly, a high level of internal consistency is evident for all HS-FOCUS domains for both patient groups (alpha, all ≥0.77; Table 3).

**Table 3:** Internal consistency at baseline and follow-up by patient type for HS-FOCUS total score and domains

		Caregiver-reported patients (3–26 years)		Self-reported patients (12–58 years)	
		Initial assessment at baseline (*n* = 25)	Follow-up at 2–4 weeks (*n* = 25)	Initial assessment at baseline (*n* = 12)	Follow-up at 2–4 weeks (*n* = 13^a^)
Cronbach’s alpha		0.89	0.86	0.94	0.92
Cronbach’s alpha with item deleted	Lower body	0.84	0.77	0.93	0.88
	Upper body	0.86	0.82	0.93	0.95
	School/work	0.91	0.88	0.92	0.89
	Activities	0.88	0.84	0.93	0.87
	Breathing	0.85	0.81	0.90	0.89

Cronbach’s alpha > 0.70 was considered as an acceptable level of internal consistency

^a^One patient had missing domain scores at initial assessment but had data for all domain scores at follow-up

Test–retest reliability was evaluated among 19 caregiver and 14 self-reported stable patients based on GIS (Table 4). The mean HS-FOCUS total scores and all domain scores indicated high test–retest reliability. For the mean HS-FOCUS total scores measured at baseline and follow-up for both patient types the ICC values were > 0.90. At the domain score level, ICC values for both patient types comparing baseline and follow-up measurements were > 0.70 in most cases.

**Table 4:** Test–retest reliability of the shortened HS-FOCUS questionnaire for HS-FOCUS total score and domains

Stable patients based on GIS score	Caregiver-reported patients (3–26 years) *n* = 19			Self-reported patients (12–58 years) *n* = 14		
	Initial assessment at baseline	Follow-up at 2–4 weeks	ICC	Initial assessment at baseline	Follow-up at 2–4 weeks	ICC
Mean (SD) total score	1.02 (0.77)	1.00 (0.69)	0.97	0.48 (0.53)	0.46 (0.49)	0.93
Lower body	0.84 (0.98)	0.95 (1.10)	0.95	0.59 (0.74)	0.48 (0.52)	0.90
Upper body	1.58 (0.89)	1.61 (0.84)	0.91	0.58 (0.6)	0.48 (0.41)	0.83
School/work	0.59 (0.48)	0.59 (0.54)	0.71	0.19 (0.53)	0.13 (0.35)	0.92
Activities	1.05 (1.1)	0.84 (0.76)	0.68	0.49 (0.65)	0.51 (0.69)	0.79
Breathing	0.92 (0.89)	0.93 (0.85)	0.86	0.52 (0.53)	0.52 (0.82)	0.73

*ICC* was determined for stable caregiver- and self-reported patients based on GIS analysis; ICC > 0.70 was considered as an acceptable level of reliability

*Abbreviations*: *GIS* Global Impression of Severity, *HS-FOCUS* Hunter Syndrome-Functional Outcomes for Clinical Understanding Scale, *ICC* intra-class coefficient, *SD* standard deviation

### Convergent and discriminant validity of the shortened HS-FOCUS questionnaire

Spearman’s rank correlations between domains of the CHQ and HUI3 instruments and total scores of the shortened HS-FOCUS instrument are shown in Table 5. A low, moderate, and high correlation was considered as < 0.3, > 0.3, and > 0.6, respectively. As HS-FOCUS measures physical functioning and daily activities of patients with MPS II, we expected the mean HS-FOCUS total score to show a moderate to high correlation with physical domains of CHQ and HUI3 instruments. Self-reported patients (12–18 years of age) completed the CHQ Child form (CHQ-CF), and caregivers completed the CHQ Parent form (CHQ-PF) on behalf of caregiver-reported patients (of any age). For both the caregiver- and self-reported versions of the shortened HS-FOCUS instrument, there were moderate to high Spearman correlations of the HS-FOCUS total score with the physical functioning, role/social–physical, and bodily pain domains of the CHQ instrument. In addition, in the caregiver-reported version of the HS-FOCUS, there was a moderate (> 0.3) correlation between the CHQ domains of behavior and general health perceptions and HS-FOCUS total score. For the self-reported version of the HS-FOCUS, there was an additional moderate correlation between the mental health domain of the CHQ instrument and HS-FOCUS total score. Across both versions of the shortened HS-FOCUS questionnaires, there was a high correlation between total scores and the HUI3 speech and dexterity domains.

**Table 5:** Convergent and discriminant validity (Spearman rho) for HS-FOCUS total scores with validated CHQ and HUI3

Instrument	Domain	Caregiver-reported patients (3–26 years) *n* = 31	Self-reported patients (12–17 years) *n* = 7	Self-reported patients (18–58 years) *n* = 13
CHQ^a^	Physical functioning	−0.83*	− 0.58	–
	Role/social–emotional (E)/behavioral (B)	−0.16	−0.80^†^ (E), –0.09 (B)	–
	Role/social–physical	−0.68*	−0.89^‡^	–
	Bodily pain	−0.51^‡^	−0.45	–
	Behavior	0.38^†^	–	–
	Global behavior	0.22	−0.29	–
	Mental health	−0.01	−0.39	–
	Self-esteem	−0.28	−0.14	–
	General health perceptions	−0.45^†^	−0.21	–
	Change in health	−0.08	−0.23	–
	Parental impact-time	−0.01	–	–
	Parental impact-emotional	−0.04	–	–
	Family activities	−0.22	−0.53	–
	Family cohesion	−0.22	−0.64	–
HUI3	Vision	−0.21	−0.29	− 0.46
	Hearing	−0.32	−0.67	− 0.10
	Speech	−0.66*	−0.84^†^	− 0.50
	Ambulation	−0.72*	0.13	−0.87^§^
	Dexterity	−0.73*	−0.87^†^	− 0.70^‡^
	Emotion	−0.42^†^	−0.65	− 0.16
	Cognition	−0.55^‡^	−0.85^†^	− 0.08
	Pain	−0.52^‡^	0.13	−0.80^‡^

Spearman’s rho significance levels: *p < 0.0001; ^†^*p* < 0.05; ^‡^*p* < 0.01; ^§^*p* < 0.001

^a^Self-reported patients (12–18 years of age) completed the CHQ Child form (CHQ-CF) and caregivers completed the CHQ Parent form (CHQ-PF) on behalf of caregiver-reported patients (of any age)

*Abbreviations*: *CHQ* Child Health Questionnaire, *HS-FOCUS* Hunter Syndrome-Functional Outcomes for Clinical Understanding Scale, *HUI3* Health Utility Index Mark 3

In general, Spearman’s rank correlations showed convergent validity of the shortened HS-FOCUS questionnaire and had moderate to high correlations with similar constructs within the CHQ physical functioning domain scores. The correlation patterns also showed discriminant validity, i.e., dissimilar constructs had a low correlation such as between HS-FOCUS total scores and HUI3 vision scale.

### Known-group validity of the shortened HS-FOCUS instrument

**Fig. 1:**
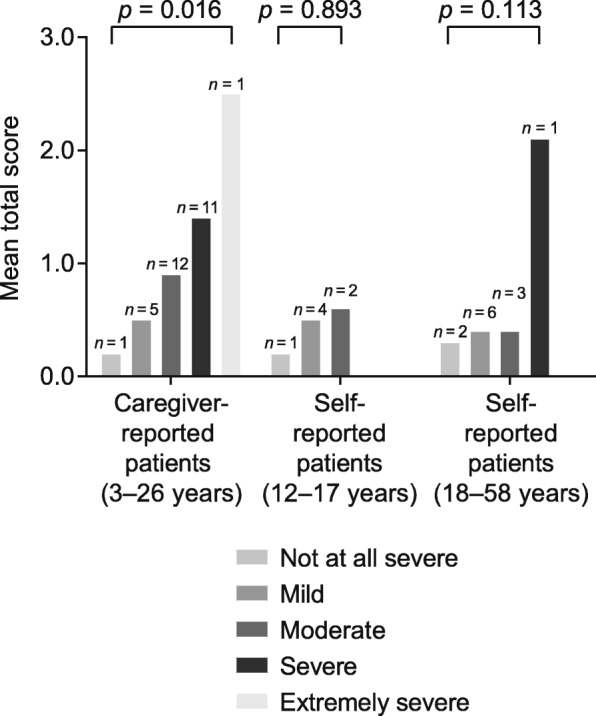
HS-FOCUS mean total scores by MPS II severity based on GIS at baseline. ANOVA was used to generate *p* values. Abbreviations: *GIS* Global Impression of Severity, *HS-FOCUS* Hunter Syndrome-Functional Outcomes for Clinical Understanding Scale, *MPS II* mucopolysaccharidosis II

The mean HS-FOCUS total scores by severity of MPS II in patients based on GIS are shown in Fig. 1. For the caregiver-reported questionnaire, there was a statistically significant difference in the mean HS-FOCUS total scores between the different severity groups (*p* = 0.016; measured by ANOVA) (Fig. 1). For the self-reported questionnaire, although mean HS-FOCUS total scores trended as expected, there was no statistically significant difference in these scores between the different severity groups (*p* = 0.893 and *p =* 0.113 for pediatric or adolescent and adult patients, respectively; Fig. 1). Post-hoc comparisons were not conducted because of the small sample sizes in this analysis.

## Discussion

Hunter Syndrome (MPS II) is a rare genetic disease with potentially devastating physical and cognitive effects. Taking into account the nature of the disease, the HS-FOCUS questionnaire was previously created as a specific tool to assess the physical functioning and activity limitations of patients with MPS II. The shortened questionnaire was designed to be a simpler and more efficient instrument to characterize the key effects of the disease in individual patients. In this prospective observational study, we validated the shortened instrument using data obtained from patients and parents/caregivers answering respective versions of the questionnaire.

Depending on the age or disease severity of patients, caregivers or patients completed the appropriate version of the questionnaire. For both groups, the most common comorbidities were carpal tunnel syndrome and cardiac disease. A large proportion (77.4%) of the caregiver-reported patients were reported to have CI. Reflecting their disease severity, mean total HS-FOCUS scores were greater for caregiver-reported patients (especially adults) than for self-reported patients, confirming higher levels of diminished functional abilities. Likewise, patients with MPS II with CNS involvement had approximately double the total scores of those without CNS involvement.

Both questionnaire versions demonstrated good internal consistency reliability and test–retest reliability. The shortened instrument also demonstrated moderate to high Spearman correlations with the physical functioning, role/social–physical, and bodily pain domains of the CHQ tool. Patients with severe MPS II have worse outcomes and diminished functional abilities, therefore it was expected that there would be differences in mean HS-FOCUS total scores for different disease severity. For caregiver-reported patients, the shortened HS-FOCUS instrument successfully detected differences in MPS II severity levels based on GIS. For self-reported patients, although a differentiating severity trend with mean HS-FOCUS total score was suggested by the data, there was no statistical difference between the different severity groups, most likely because of low patient numbers. Post-hoc comparisons between individual severity levels were not conducted because of low sample sizes.

Similar correlations to those found in our study were reported in the previous study of the shortened HS-FOCUS that was based on data derived from a Phase II/III trial [2]. Both the patient- and caregiver-reported versions of the original full-length HS-FOCUS instrument demonstrated good internal consistency and test–retest reliability (alpha > 0.70 and ICC > 0.70) for all domains except breathing, sleeping, and schooling/work, and construct validity showed moderate to high correlations with CHQ and HUI3 in activity-related concepts [13]. Wilkund et al. [2] found a high Spearman correlation between the original HS-FOCUS [13] and the subsequently developed shortened version. The shortened HS-FOCUS also demonstrated internal consistency and test–retest reliability (alpha > 0.70 and ICC > 0.70) for most domains except breathing and school/work and concurrent validity (correlation > 0.30) with similar concepts of previously validated tools [2]. Data for those studies, however, were collected in a 53-week placebo-controlled multinational trial involving patients (NCT00069641) with the attenuated form of MPS II where respondents provided direct responses to the original full-length HS-FOCUS [2, 13].

Limitations of the study reported here include the non-clinical determination of the presence of CNS involvement and its severity that relied upon the responses of patients or caregivers. One patient who completed the self-reported questionnaire characterized himself as having moderately severe CI in spite of the criteria for completing the self-reported questionnaire (ability to understand and provide written responses to the questions). In addition, the study had a small sample size and unbalanced groups. However, these factors are directly related to the rarity of MPS II. Small sample size also was found to be a limitation in another published quality-of-life study of patients with MPS II [16], and for studies in rare diseases in general [17, 18].

This 1-month study did not include any patient intervention and a change in patients' disease status was not expected, and therefore, the study did not allow for the tool’s responsiveness to changes in physical functioning and activity limitations to be evaluated. Responsiveness of the tool would be better evaluated in a treatment setting of longer duration where patients have the opportunity to experience changes to their disease state.

## Conclusions

Both the caregiver- and self-reported versions of the shortened HS-FOCUS questionnaire demonstrated a high degree of reliability, even within the small sample sizes of this study. The shortened HS-FOCUS questionnaire demonstrated good convergent and discriminant validity through Spearman rank correlations. The shortened HS-FOCUS differentiated between GIS-score defined severity groups for patients with MPS II. The tool’s responsiveness to changes in physical functioning and activity limitations was not evaluated in this study because of its short duration and the fact that this was a naturalistic, observational study without intervention, meaning that patients were relatively stable in their disease state for the duration of the study.

Overall, the results confirm that the shortened HS-FOCUS questionnaire is a valid and reliable tool to assess the physical functioning and activity limitations impact of MPS II. The shortened version of the instrument should reduce respondent burden compared with the full original HS-FOCUS tool. This study affirms the suitability of the shortened HS-FOCUS instrument for the assessment of the physical functioning of patients with MPS II, which could be used for this purpose in future clinical trials or observational studies.

## Abbreviations

ANOVA: Analysis of variance
CHQ: Child Health Questionnaire
CI: Cognitive impairment
CNS: Central nervous system
GIS: Global Impression of Severity
HS-FOCUS: Hunter Syndrome-Functional Outcomes for Clinical Understanding Scale
HUI3: Health Utility Index Mark 3
ICC: Intraclass correlation coefficient
MPS II: Mucopolysaccharidosis type II
PRO: Patient-reported outcome
